# *Via media*: Role and responsibilities of the independent safety officer

**DOI:** 10.1017/cts.2019.393

**Published:** 2019-08-29

**Authors:** M. E. B. Holbein, Barbara N. Hammack, Ann J. Melvin, Tamsin A. Knox

**Affiliations:** 1 Department of Population and Data Science, University of Texas Southwestern Medical Center, Dallas, TX 75390, USA; 2 Colorado Clinical & Translational Sciences Institute, University of Colorado Denver – Anschutz Medical Campus, Aurora, CO 80045, USA; 3 Division of Pediatric Infectious Disease, Department of Pediatrics, University of Washington and Seattle Children’s Research Institute, Institute of Translational Health Sciences, Seattle, WA 98105, USA; 4 Department of Public Health and Community Medicine, Tufts Clinical and Translational Science Institute, Tufts University School of Medicine, Boston, MA 02111, USA

**Keywords:** Safety, monitoring, safety officer, risk, clinical trials as topic, conflict of interest, trial oversight, humans

## Abstract

Every research study that includes volunteer participants requires safety assurances in proportion to the risks of the study. Investigator-initiated clinical research can present unique regulatory challenges particularly for studies with a risk profile that warrants more oversight than minimal risk but less than for large, commercial, or high-risk research. The use of an independent safety officer (ISO) offers a middle way of right-sizing oversight to match the risk. ISOs are clinicians or researchers with relevant expertise who are independent of the investigator and the research study. Their relationship to the study is defined by a formal charter which is aligned with the protocol and Data and Safety Monitoring Plan to address the oversight process, responsibilities of the ISO, and clearly describe the variables to be monitored. The ISO responsibilities include reviewing safety data, adverse events, recruitment, demographics, study progress, data quality, protocol changes, and any new scientific information that pertains to the trial. Finally, the ISO reports in their review on any significant findings may propose modifications to the study or a need to stop the trial.

## Introduction

Academic health centers (AHCs) are frequently the site of investigator-initiated clinical research (IICR). These are clinical studies that an individual researcher designs and conducts. They are neither initiated nor overseen, but may be funded, by a commercial enterprise. The scope can include single as well as multi-site investigations and can be part of a collaborative or cooperative group. Large cooperative group trials, such as those assembled by National Institutes of Health (NIH) institutes, have multiple sites with safety monitoring built into their structure and usually include a dedicated safety committee. Single-site IICRs frequently have relatively limited numbers of subjects, are undertaken in less clinically complex populations, have simpler protocols, or include moderate to minimal risk interventions. Thus, many such trials warrant a simplified approach to safety oversight. Often, this entails investigator-led data integrity and safety monitoring to assure that the study meets established regulatory and ethical standards. However, not infrequently, additional oversight is warranted.

Safety surveillance of a clinical study should be designed specifically in proportion to the risk. Even the simplest trials require a basic plan to assure meeting not only federally mandated regulatory standards but also ethical standards of subject protection [1,2]. Many factors define the inherent “riskiness” of a trial. Considerations include the number of subjects, the clinical complexity or risk of the intervention, specific concerns of a study site, the experience of the investigator, or the characteristics of the study population. All of these contribute to the risk and an assessment of the need for oversight. Institutional review boards (IRB) usually require a formal written Data and Safety Monitoring Plan (DSM Plan) for assuring that human subjects are protected. Part of such a plan includes monitoring and safety assurances in proportion to the assessed risk to the human subjects enrolled. Sometimes additional safety assurances beyond solely investigator-led monitoring may be needed for a more comprehensive plan. Such trials do not warrant the oversight and advice of a traditional Data and Safety Monitoring Board (DSMB) [2,3] but have one or more characteristics that warrant an increased level of oversight to assure participant safety. Therefore, an intermediate level of oversight is needed.

The usual approach to this “in between” level of oversight is to engage an investigator not involved with the trial to provide an independent review of the data and safety assurances during the trial. This individual is an independent safety officer (ISO). This *via media*, or middle way, approach to participant safety is generally recognized [4], but the roles and responsibilities are not well characterized in the literature. For this discussion, the intent is to address the scenario with a single-site, investigator-initiated, moderate risk trial without externally appointed monitors. We present a comprehensive approach to describing the roles, responsibilities, processes, and tools needed for effective oversight by an ISO.

## Role of an ISO

### Characteristics of a Trial That May Require an ISO

Even single-site trials may have an increased risk profile that justifies an ongoing, independent evaluation. Many of these trials will be of sufficient complexity and/or risk to benefit from a full DSMB or a safety monitoring committee [5]. However, for low and moderate risk single-site trials, monitoring by an ISO provides an alternative monitoring option. Examples include clinical trials involving Food and Drug Administration (FDA)-approved drugs used in unapproved indications or populations, non-significant risk medical devices, nutritional products used as a drug, research-only interventions such as an insulin clamp, or behavioral interventions with the possibility of psychological adverse events (AEs). ISOs may also be appropriate for higher risk single-site trials of short duration, such as pilot studies, for which convening a full DSMB is not feasible. Characteristics of trials for which monitoring by an ISO is appropriate are detailed in Table 1.

**Table 1.** Characteristics of research typically monitored by an ISO

Trial characteristic	Examples
Type	Investigator-initiated single site Potential for public scrutiny
Population	Vulnerable population (children, pregnant women, prisoners, and people with dementia or psychiatric conditions) Ethnic minorities (language barrier, need for cultural sensitivities) Diseases or conditions with possibility of poor outcome Unusually large number of participants for a single-center trial Healthy volunteer participants exposed to moderate risk interventions
Design	Interventional (e.g. behavioral, use of drugs outside FDA-approved labeling, use of placebo) Complex clinical protocol (increased likelihood of deviations) Blinding/masking Novel technology Non-significant risk devices (without FDA-approved labeling) Pilot studies
Investigator	Junior investigator Investigator with prior research regulations noncompliance

ISO, independent safety officer.

### What Is an ISO?

We chose the term “independent safety officer” for this review to highlight the broad function of this individual which is inclusive of not only adjudicating medical (clinical) events but also the conduct of the trial and oversight of data. The role of an ISO is distinct from that of a medical safety monitor used by some NIH institutes, or medical officers used by pharmaceutical companies, whose primary role is to monitor serious adverse event (SAE) reports for multicenter trials. For example, the National Institute of Neurological Disorders and Stroke defines a “medical safety monitor” as having independent surveillance in the setting of a multi-site trial of the “ongoing monitoring of reports of SAEs submitted by the clinical centers in real time to ensure good clinical practice and to identify safety concerns quickly” [6]. Other terms frequently used for the broader monitoring role of the ISO include independent medical monitor [6] and independent safety monitor [7].

As implied by the name, the ISO is independent of the clinical trial that they are monitoring. They are not part of the study team. An ISO is usually a physician or investigator with experience and training in the disease or condition as well as in the intervention that is being studied. Relevant experience is essential in order to be able to evaluate not only the progress and quality of a trial but also, more importantly, a contemporaneous assessment of the safety. Typically, this would be an individual with relevant research experience. Other areas of expertise may be indicated, such as experience in pediatrics for a trial involving children.

### Commissioning an ISO

Several factors should guide the selection of an ISO for a trial. In addition to independence from the study, the ISO should not have an interest in the potential study outcome, scientific, professional, financial, or otherwise, that could impact their decision-making. Frequently, ISOs are individuals from the investigator’s institution and, due to the clinical overlap, may be from the investigator’s home department or division. However, the ISO should not have a relationship with the investigator that could result in a conflict of interest (COI). For example, the ISO should not be a supervisor or subordinate of the investigator or a current scientific collaborator. Most AHCs have policies for COI declarations that can assure additional independence with regard to financial interests as well [8]. The ISO should declare any potential COI and sign a COI statement prior to the initiation of the trial. The ISO should have the time to perform this function and be available for the duration of the trial. In addition to the requisite expertise and independence, an additional qualification is availability and in most cases, a willingness to serve in the role without financial compensation since many IICR trials are small and marginally funded. Frequently the trial sponsor, such as the NIH institute program officer, will request to approve the ISO selected by the investigator to monitor the trial.

## Responsibilities of the ISO

### Inclusion of an ISO in the DSM Plan

IRBs require a description of the safety monitoring process for the study, called a DSM Plan. The DSM Plan formalizes how the safety of the subjects and the validity of the data will be maintained during the trial and includes a description of the individuals responsible for monitoring the overall investigation. The use of an ISO should be included and described in the DSM Plan. The DSM Plan typically includes the aspects of the trial that will be reviewed, the frequency of data review and written reports, plan for AE identification and reporting, plan for monitoring of data quality and accuracy, and the criteria for decision-making regarding continuation, modification, or termination of individual participants or the clinical study if applicable. Details on the role of the ISO in fulfilling the DSM Plan are provided in the ISO charter.

### Writing an ISO Charter

An ISO charter is a guiding document, carefully aligned with the research protocol and statistical analysis plan that should be developed by the primary investigator (PI) collaboratively with the ISO prior to any participant enrollment [5]. Several revisions may be needed to accomplish a mutually agreed upon charter. The predetermined guidelines that address the oversight process should clearly define the responsibilities of the ISO, including the purpose, frequency, and structure of meetings, the data to be reviewed, and the content of the reports as shown in Table 2.

**Table 2.** Key elements of an ISO charter

Unique study identification (title, document identifiers, and IRB number) Trial overview – study design and protocol summary Responsibilities and functions of the ISO a. Review criteria for safety b. Detail the data variables to be provided by the investigator and reviewed by the ISO c. Specify the frequency of monitoring d. Overview of the process for review of AEs ISO meetings a. Meeting attendees b. Frequency of scheduled meetings c. Meeting format Study stopping criteria ISO reports Amendments to the ISO charter Attachments (including data tables, confidentiality statement, and COI statement)

ISO, independent safety officer; IRB, institutional review board; COI, conflict of interest; AEs, adverse events.

An ISO charter lays out the responsibilities of the ISO for safety and data monitoring. It includes a study overview and the criteria for assessing safety. The charter specifies the study data needed to confirm safety assessments, adjudicate the relation of AEs to study participation, and confirm that AE reporting is complete. In addition, it also considers the types of data needed to achieve primary and secondary outcomes. Data tables should be compilations of cumulative data from individual subjects to allow a review of combined primary data while preserving participant confidentiality. Finally, the charter specifies the way to modify the charter during the course of the study, if needed. These amendments and any attachments such as the COI form will appear in the charter appendix.

Confidentiality should be maintained throughout the monitoring process. Therefore, the ISO should commit to maintaining trial confidentiality and should not receive identifiable patient health information nor share trial data. The charter can include a confidentiality statement or a separate document should be signed prior to the start of the trial.

### Activities Prior to Trial Initiation

Prior to trial initiation, the ISO should review relevant trial documents and suggest modifications if appropriate. The ISO should also review and approve the content and format of the study reports as well as proposed study stopping rules and unblinding procedures, if applicable. If possible, this should be done before submitting to the IRB for review and approval to avoid delays. The ISO should receive a complete set of reference documents, as listed in Table 3, from the study team. Some granting agencies will want a letter from the ISO documenting their agreement to serve in this function for the duration of the trial and a copy of their *curriculum vitae*. These documents are typically kept in a real or virtual Regulatory Binder.

**Table 3.** Documents needed by the ISO prior to trial initiation

Regulatory Binder for trial documents (protocol, informed consent documents, statistical analysis plan, ClinicalTrial.gov registration number, IND or IDE information) Charter Data and safety monitoring plan Signed COI statement Signed confidentiality agreement Letter to grant agency confirming the ISO position, if needed Projected meeting dates for the first year Data collection tools

IND, Investigational New Drug application; IDE, Investigational Device Exemption application; ISO, independent safety officer; COI, conflict of interest.

A particular difficulty involves decisions to stop a study for safety, efficacy, or futility. If the study has early stopping principles for efficacy or futility, this should be stated in the charter with clear cut-off criteria. The ISO, the PI, and the study biostatistician should discuss these targets and the time-line for interim analyses, if applicable. It is important that all the study team members are in agreement as to what triggers cessation of a study. The charter should also include procedural details for unblinding the study results in case safety or efficacy targets are met. Equally important is a definition for futility. This includes the possibility of stopping a trial whose enrollment will not meet targeted number of enrollees within a reasonable amount of time.

It is also helpful for the ISO to have a study start-up meeting with the study team prior to trial initiation. This ensures that the study team knows how to reach the ISO and there is agreement on how AEs and SAEs should be reported to the ISO in terms of specific documents (IRB reports, e-mails) and the time frame for these reports. A problem can arise with questions of how long AEs such as deaths are to be reported after study participation ends. Again, this should be clarified with the protocol and IRB at study initiation. At the initial meeting, the ISO can agree on projected meeting dates for the first year of the study by enrollment, time frame, or study strata such as a dose escalation study.

Finally, the ISO will want to review data collection tools in coordination with the study data manager or study coordinator. If the study does not have a data manager, the study coordinator may need additional assistance in developing and using data collection tools for the ISO which are cumulative rather than the individual participant data entry forms used in the study. The ISO, in particular, will want a cumulative set of AEs and SAEs by date and type of event which will make oversight more accurate and improve reporting.

### Content for Meetings, Reports, and Reviews

It is important that the data submitted for review by the ISO be as current, complete, and accurate as possible. Prior to the first data and safety review meeting, the ISO will need to ensure that the study team can provide complete cumulative data. Initially, this can take several iterations for the study team to present comprehensive and cumulative data from individual participant records. The data should be submitted to the safety officer approximately 1 week before the scheduled meeting. The data sent for review should include (1) safety review: data tables with laboratory values and other measurements, AEs; (2) trial conduct: recruitment strategy, recruitment and enrollment statistics, disqualified and excluded individuals, protocol deviations, study progress timeline, study progress by participant, summary statistics, newly published relevant data, and major protocol changes; and (3) data quality: procedures for data quality control and adherence monitoring.

Meetings of the PI and the ISO are scheduled at predetermined intervals as stated in the charter. In a higher risk trial, this may occur after each participant is enrolled. Other common times for review are (1) based on time intervals such as quarterly; (2) based on enrollment rates such as after the first 10 participants or after the first 25% of participants are enrolled; or (3) after each group or cohort of a study is enrolled and before dose escalation. The PI should meet with the ISO a minimum of once annually. It should be noted that since the primary role of the ISO is participant safety and study conduct, the ISO reviews all SAEs and unanticipated problems (UPs) in real-time as well, including patient-level data, if indicated. This may necessitate an unscheduled meeting if significant events have occurred.

The PI or ISO will prepare the agenda for the meeting. After the meeting, the ISO and/or study coordinator will prepare the minutes. These should include a brief synopsis of all data evaluated, including SAEs and AEs, if any. The ISO usually is responsible for the final written report with recommendations to the PI for any suggested changes in study procedures. Future meeting dates may be adjusted based on safety events, enrollment success, and any problems with data collection. At subsequent meetings, prior recommendations should be reviewed to be sure appropriate changes were made in the study procedures. If an interim analysis is needed or planned (for efficacy, safety, or futility), the ISO will work with the biostatistician for statistical analysis of the data.

The role of the ISO in determining whether the study should continue as planned, proceed with modifications, or be terminated should also be clarified prior to the initiation of the trial. After the meeting, the ISO will prepare a summary for submission to the PI. This report will provide comments on the data reviewed, describe study safety, progress, and performance, discuss any concerns or suggestions for modification, and provide recommendations as to the safety, continuation, modification, or early termination of the study [5].

### Adjudicating AEs

The charter specifies who is to adjudicate AEs, the PI or the ISO. If the PI determines whether an AE is related to study participation, the ISO, in reviewing the event, should confirm this separately in their review of the event. In the event of a disagreement which cannot be resolved by discussion, the ISO may want to involve a third expert for opinion. The definitions of a SAE and an UP generally conform to federal standards [1,9]. The ISO should also see all follow-up reports of SAEs as these may be more informative about the event and ensure participant safety.

Reporting SAEs depends on the IRB of record. Some IRBs only require reporting SAEs if an event requires a change in the study protocol. UPs must always be reported. While AEs that do not meet the criteria for SAEs or UPs do not need to be reported, they should be reviewed by the ISO. These events may be sentinel events that point out problems with the study intervention or study protocol which can be modified to reduce participant risk or may indicate that closer monitoring is needed. For example, if AEs occur more frequently in participants with a history of asthma, these participants can be monitored more closely.

It is helpful for the ISO to keep a list of AEs, dates, and follow-up reports. Documentation of this can be included in the periodic meeting reports. Not all events need immediate action.

### Evaluating or Enforcing Stopping Rules

Many IICR studies do not have stopping rules. However, in those that do, evaluating outcomes based on stopping rules can be one of the most difficult tasks for an ISO. This is considerably easier if close attention is paid before a study starts to the definitions of stopping rules and agreement in understanding these rules between the PI, biostatistician, and the ISO.

If stopping rules are met, the ISO has an obligation to recommend stopping a study. It is also possible to suspend enrollment to the study while detailed statistical analysis is carried out and to allow time for any further SAEs to appear. The ISO should document the reason for suspending or halting enrollment supported by the statistical analysis in the report sent to the PI which should also be sent to the IRB and the grant agency.

Concerns about safety or interim efficacy analyses may require unblinding of the study data. In this case, all efforts should be made to keep the PI and study staff blinded. The unblinding may be done by the data manager or an independent biostatistician who is not involved in the study. Anyone seeing the unblinded data has a responsibility to maintain confidentiality.

## When More Expertise Is Needed

Occasionally, either due to the complexity of a trial or to other technical attributes, additional expertise beyond the ISO is needed to accomplish a comprehensive review. With additional personnel, this is considered a “Safety Committee.” Most frequently, a biostatistician will be the additional reviewer. This may be the same individual that served as the biostatistician who contributed to the design of the trial and who may also be part of the data collection team. If unblinded analyses are envisioned for safety or for early stopping principles, a separate independent biostatistician, other than the study biostatistician, should be included in the Safety Committee. Other additions can include a content expert for nonclinical issues, a methodology consultant, or another clinician who has additional specific expertise. Such two- or three-member committees are constituted in a manner similar to a single individual complete with a charter, defined roles, reviewing and reporting responsibilities. However, the complexity of the process, both formal and informal, is substantially less than for a DSMB.

## Conclusion

The role of an ISO is underutilized in monitoring investigator-initiated clinical studies. Commissioning an ISO provides a middle way, a *via media*, between monitoring solely by the PI and monitoring by a larger entity such as a DSMB. An ISO is particularly useful in monitoring smaller, single-site studies of low to moderate risk without the scheduling difficulties and costs of a full DSMB. An ISO should have prior clinical research experience as well as expertise in the specific research area. Central to the function of an ISO is a clear DSM Plan and a written charter which guides the monitoring, and available options should safety issues arise. In addition, an ISO provides important support and guidance for junior investigators both in supervision for safety and for data quality. There is minimal literature about the important role an ISO can serve in an AHC and in institutes supported by the NIH. This article outlines the role of an ISO, their interactions with the PI and study team, and their responsibilities with respect to safety oversight and study conduct. With this guidance, an ISO can provide support to the PI and enhance safety for study participants.
